# Supplementary material for: Direct investigation of the reorientational dynamics of A-site cations in 2D organic-inorganic hybrid perovskite by solid-state NMR
Source: Nat Commun. 2022 Mar 21;13:1513. doi: 10.1038/s41467-022-29207-6 (PMC8938534; doi:10.1038/s41467-022-29207-6)
Supplement: Supplementary file 1 — Supplementary Information [file 41467_2022_29207_MOESM1_ESM.pdf]

Supplementary Information (SI)

Direct Investigation of the Reorientational Dynamics of A-site Cations in 2D Organic-Inorganic Hybrid Perovskite by Solid-State NMR

Cheng-Chieh Lin[1,2], Shing-Jong Huang[3], Pei-Hao Wu[4], Tzu-Pei Chen[5], Chih-Ying Huang[1,2], Ying-Chiao Wang[5], Po-Tuan Chen[6], Denitsa Radeva[7], Ognyan Petrov[7], Vladimir M Gelev[7], Raman Sankar[8], Chia-Chun Chen[9], Chun-Wei Chen[1,5,10], Tsyr-Yan Yu[1,4]**

[1]International Graduate Program of Molecular Science and Technology (NTU-MST), National Taiwan University, Taipei 10617, Taiwan
[2]Molecular Science and Technology Program, Taiwan International Graduate Program (TIGP), Academia Sinica, Taipei 11529, Taiwan
[3]Instrumentation Center, National Taiwan University, Taipei 10617, Taiwan
[4]Institute of Atomic and Molecular Sciences, Academia Sinica, Taipei 10617, Taiwan
[5]Department of Materials Science and Engineering, National Taiwan University, Taipei 10617, Taiwan
[6]Department of Vehicle Engineering, National Taipei University of Technology, Taipei 10608, Taiwan
[7]Department of Chemistry and Pharmacy, Sofia University, 1 James Bourchier Boulevard, 1164 Sofia, Bulgaria
[8]Institute of Physics, Academia Sinica, Taipei 115201, Taiwan
[9]Department of Chemistry, National Taiwan Normal University, Taipei 11677, Taiwan
[10]Center of Atomic Initiative for New Materials (AI-MAT), National Taiwan University, Taipei 10617, Taiwan

Corresponding Authors
E-mail: chunwei@ntu.edu.tw (CWC) and tyyu@pub.iams.sinica.edu.tw (TYY)

Supplementary Methods

Samples preparation

Synthesis of [U-^{13}C, 99%;U-^{15}N, 98%] methylammonium iodide. Potassium ^{15}N-phthalimide can be purchased (e.g. from Cambridge Isotope Laboratories) or made as follows: 4.0 g NaOH (100 mmol) and 5.5 g $^{15}NH_4Cl$ (Cortecnet, France, 98% ^{15}N, 100 mmol) were suspended in 100 ml 96% ethanol in a tightly stoppered 250 ml round bottom flask and stirred for 24 h at room temperature. 13 g phthalic anhydride (88 mmol, Fluorochem, UK) was added and the mixture was stirred for an additional 3 days. A distillation head was attached to the flask and the ethanol was distilled off completely. The flask was heated to approximately 300°C until the product started to sublimate on the upper walls of the flask. Heating was continued for several hours until 12 g of ^{15}N-phthalimide (82 mmol, 82% from $^{15}NH_4Cl$) was collected as large yellowish crystals. 3.16 g (21.5 mmol) ^{15}N-phthalimide and 8.3 g K_2CO_3 (60 mmol) were suspended in 20 ml DMF (dried over 3Å molecular sieves). Alternatively, 4.00 g ^{15}N-potassium phthalimide (21.5 mmol, 98% ^{15}N, Cambridge Isotope Laboratories), 1.38 g K_2CO_3 (10.0 mmol) were suspended in 20 ml DMF (dried over 3Å molecular sieves). To this suspension was added dropwise 3.50 g $^{13}CH_3I$ (24.5 mmol, 99% ^{13}C, Cambridge Isotope Laboratories). The reaction mixture was stirred while protecting from light for 3 days. The salts were filtered off and the DMF solvent was removed under reduced pressure. The resulting solids were suspended in ethyl acetate and filtered. The filtrate was extracted with saturated NaCl solution, dried with Na_2SO_4 and the solvent was removed under reduced pressure to yield 3.0 g ^{13}C-methyl-^{15}N-phthalimide (19 mmol, 87%). This was suspended in 50 ml methanol and 1.13 ml (1.2 equivalents) of 80% hydrazine monohydrate was added. The solution was boiled under reflux for 2 hours. 12 ml 6M HCl and 5 ml H_2O were added, resulting in a thick white suspension, which was stirred overnight. The suspension was filtered, and the solid was washed twice with 25 ml of water. The combined filtrate and washes were reduced under vacuum to 50 ml and filtered again. 10 ml of 10M NaOH was added and the basic solution was purged with a gentle stream of argon while heating in a 100°C oil bath. The evolving methylamine was recaptured by bubbling the gas stream through 50 ml of 5% HI. Purging was continued for 5 hours until the smell of amine disappeared from the NaOH solution. (The NaOH solution can be checked for residual methylamine by ^{13}C-NMR.) The HI solution was evaporated under reduced pressure and the resulting orange solid was dissolved in methanol, precipitated with diethyl ether, and filtered. The methanol/ether precipitation was repeated two more times. Drying the product *in vacuo* over P_2O_5 yielded 2.1 g of fine white crystals of $^{13}CH_3{}^{15}NH_3I$ (13 mmol, 60% from the potassium phthalimide). The associated ^{1}H NMR (500 MHz) and ^{1}H-decoupled ^{13}C

NMR (125 MHz) spectra are shown in Supplementary Fig. 9 and Supplementary Fig. 10, respectively.

Details of materials characterizations

The photoluminescence (PL) spectra were recorded with a home-built laser scanning confocal microscope with 372 nm pulse laser (PicoQuant). Optical absorption spectra were measured by a microscopy system with an Ocean Optics spectrometer. The structure and phase purity of the 2D organic-inorganic hybrid perovskite crystals were characterised by powder X-ray diffraction (PXRD) patterns using a Bruker D8 with Cu-Kα radiation (40 kV, λ = 1.5406 Å). The temperature-dependent PXRD measurement using a Bruker D8 Discover X-ray diffraction system with Cu-Kα radiation in Bragg-Brentano geometry and equipped with a temperature control system (77-350K). The examination of possible phase transitions in 2D organic-inorganic hybrid perovskite crystals was investigated using Differential Scanning Calorimetry (DSC). The DSC heating curves were collected using a TA Q200 thermal analysis instrument at a scan rate of 5K min^{-1} and heated from 225 K to 300 K in sealed aluminum pans under ambient conditions.

Theory of REDOR analysis

Under magic angle spinning condition, the dipolar coupling between an A-X heteronuclear spin pair can be expressed as follows

$$\omega_D(\alpha,\beta;t) = \frac{D}{2}\left[\sin^2\beta\cos(2\alpha + 2\omega_r t) - \sqrt{2}\sin 2\beta\cos(\alpha + \omega_r t)\right], \qquad (1)$$

where $D = \frac{\mu_0\hbar}{4\pi}\frac{\gamma_A\gamma_X}{r_{A-X}^3}$; ω_r is the rotor speed in rad/s; μ_0 is the vacuum permittivity; ħ is the reduced Planck's constant; γ_A and γ_X are the gyromagnetic ratios of spin A and spin X, respectively; r_{A-X} is the internuclear distance; α and β are the azimuthal and polar angles describing the orientation of the internuclear vector in the rotor frame, respectively. Using the average Hamiltonian theory, the averaged dipolar coupling over one rotor period is zero. For a typical REDOR NMR experiment, Fig. 2a, the inverting 180° pulses applied in the dephasing channel result in the non-zero dipolar coupling under MAS. Quantitatively, the averaged recoupled dipolar coupling per rotor cycle can be calculated as follows

$$\overline{\omega_D}(\alpha,\beta) = \pm\frac{1}{T_r}\left\{\int_0^{T_r/2}\omega_D(\alpha,\beta;t)\,dt - \int_{T_r/2}^{T_r}\omega_D(\alpha,\beta;t)\,dt\right\} =$$

$$\pm\frac{\sqrt{2}D}{\pi}\sin 2\beta\sin\alpha \equiv \pm 2\sqrt{2}\upsilon_{D,rigid}\sin 2\beta\sin\alpha, \qquad (2)$$

where $\nu_{D,rigid}$ is the dipolar coupling in Hz unit and "rigid" refers to the absence of molecular reorientation of the ^{13}C-^{15}N vector with respect to the rotor frame. Here, we consider the $^{13}C\{^{15}N\}$ REDOR dephasing of $^{13}C,^{15}N$-MA in 2D perovskite crystal, where the ^{13}C and ^{15}N nuclear spins of $^{13}C,^{15}N$-MA are covalently bonded and the internuclear distance is fixed. In the presence of the reorientational motion of the ^{13}C-^{15}N vector, one may relate an order parameter $\mathcal{S}$ linearly with the motional averaged dipolar coupling, υ_D, to

$$\mathcal{S} = (\upsilon_D/\upsilon_{D,rigid}), \qquad (3)$$

since both the $\mathcal{S}$ and ν_D parameters share the same degree of randomness averaged by the same reorientational motion.[1,2] Here, we have $\mathcal{S} = 0$ for completely random reorientational motion of the ^{13}C-^{15}N vector, $\mathcal{S} = 1$ for the absence of reorientation motion of the ^{13}C-^{15}N vector. The associated dipolar-dephasing phase accumulation for N rotor cycles period can be calculated as follows

$$\Delta\varphi(\alpha,\beta;N) = \upsilon_D\cdot NT_r = \pm 2\sqrt{2}\cdot\mathcal{S}\cdot\upsilon_{D,rigid}\cdot NT_r\cdot\sin 2\beta\sin\alpha, \qquad (4)$$

and the REDOR dephasing, $\Delta S/S_0$, for a powdered 2D perovskite sample after N rotor cycles can be calculated as follows

$$\frac{\Delta S}{S_0} = 1 - \frac{1}{4\pi}\int_{\alpha=0}^{\alpha=2\pi}\int_{\beta=0}^{\beta=\pi} d\alpha\sin\beta\,d\beta\cos\left[2\sqrt{2}\cdot\mathcal{S}\cdot\upsilon_{D,rigid}\cdot NT_r\cdot\sin 2\beta\sin\alpha\right]$$

$$= 1 - \frac{\pi}{2\sqrt{2}}J_{\frac{1}{4}}\left(\sqrt{2}\cdot\mathcal{S}\cdot\upsilon_{D,rigid}\cdot NT_r\right)J_{-\frac{1}{4}}\left(\sqrt{2}\cdot\mathcal{S}\cdot\upsilon_{D,rigid}\cdot NT_r\right), \qquad (5)$$

where J is the Bessel function of the first kind.

1. Supplementary Figures

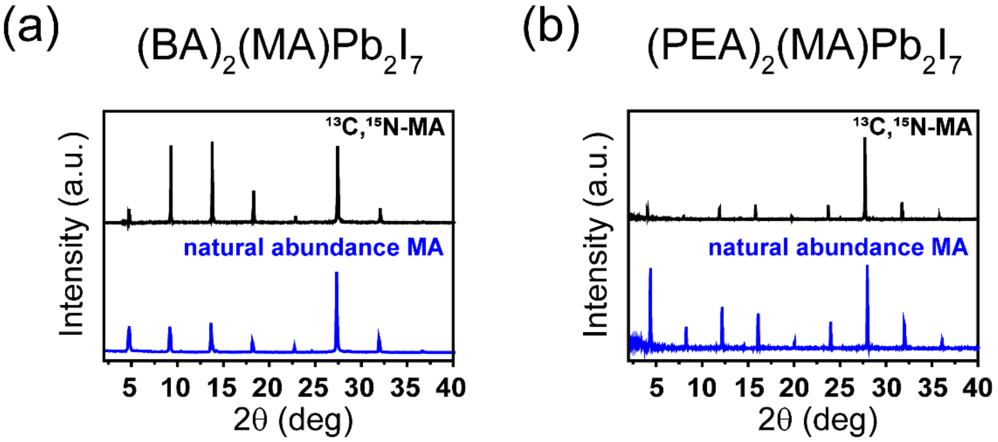

Supplementary Fig. 1 The structural characterisations of 2D OIHP (n = 2) crystals using PXRD. The PXRD spectra of (a) 2D $(BA)_2(MA)Pb_2I_7$ (n = 2) and (b) 2D $(PEA)_2(MA)Pb_2I_7$ (n = 2) crystals, synthesised with both natural-abundance MA (blue) and $^{13}C,^{15}N$-MA (black), respectively.

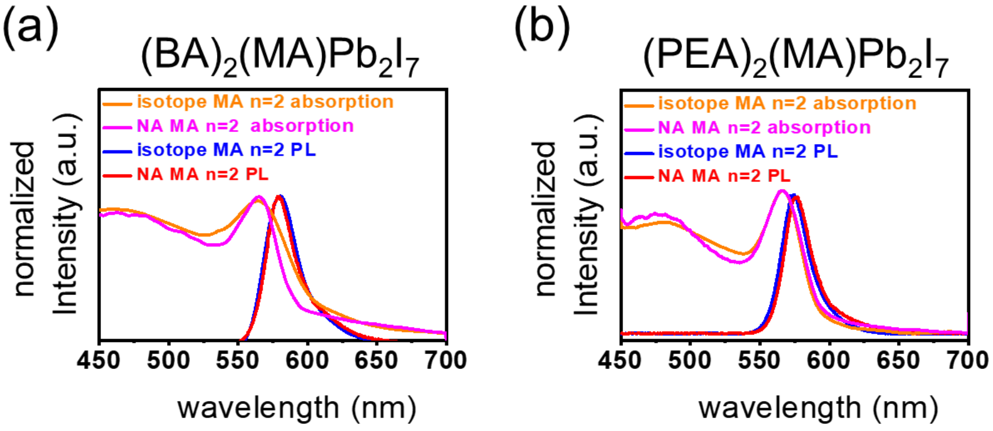

Supplementary Fig. 2 The characterisations of the optoelectronic properties of 2D OIHP (n = 2) crystals using both photoluminescence and absorption spectroscopy, respectively. The photoluminescence and absorption spectra of (a) 2D $(BA)_2(MA)Pb_2I_7$ (n = 2) and (b) 2D $(PEA)_2(MA)Pb_2I_7$ (n = 2) crystals. $^{13}C,^{15}N$-MA with n = 2 absorption spectrum: orange; natural-abundance MA with n = 2 absorption spectrum: magenta; $^{13}C,^{15}N$-MA with n = 2 PL spectrum: blue; natural-abundance MA with n = 2 PL spectrum: red)

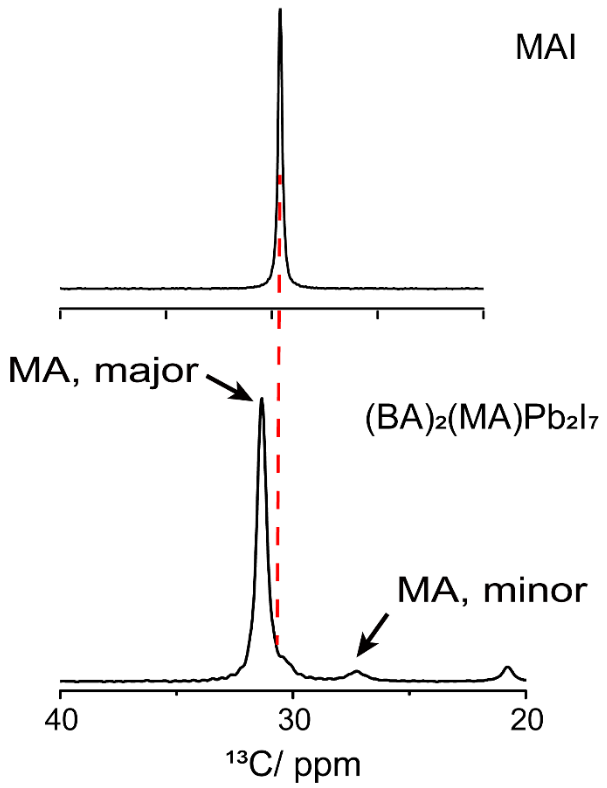

Supplementary Fig. 3 The ^{13}C CPMAS NMR spectra of ^{13}C,^{15}N-MAI crystal powder (top) and 2D $(BA)_2(^{13}C,^{15}N\text{-}MA)Pb_2I_7$ (n = 2) (down), respectively. The ^{13}C resonance peak of MAI is marked with the red dashed line to aid the comparison of the chemical shifts of the MAI crystal powder and the minor MA component.

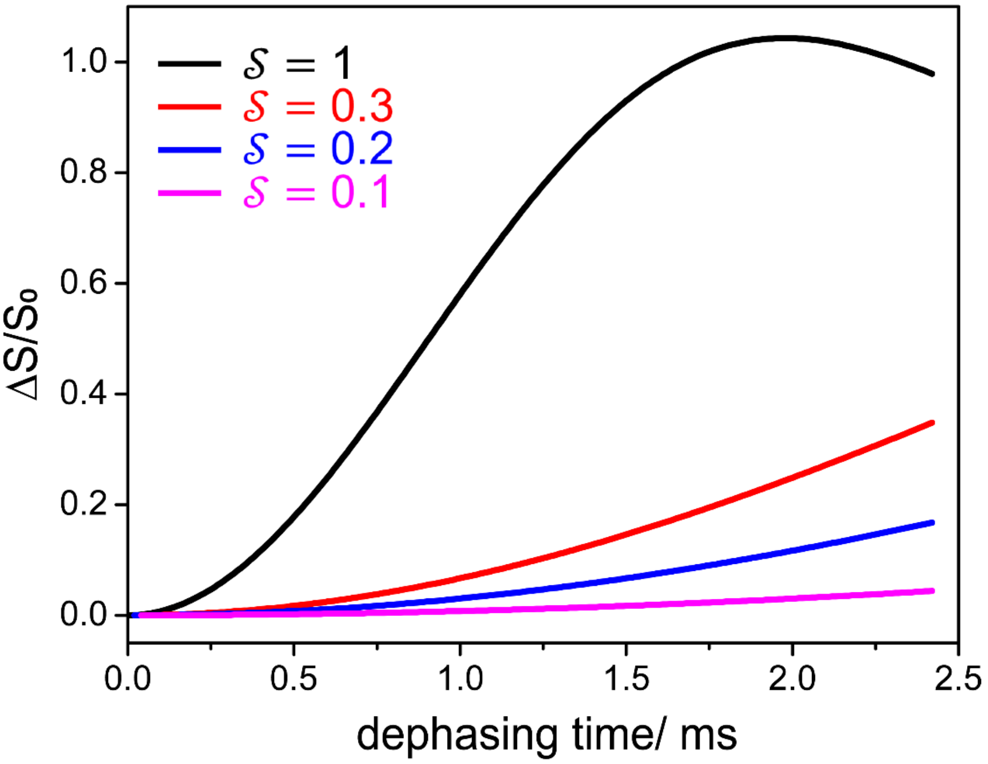

Supplementary Fig. 4 The simulated $^{13}C\{^{15}N\}$REDOR dephasing curves of $^{13}C,^{15}N$-MA cation undergoing different degrees of reorientational motion, represented by different order parameter $\mathcal{S}$ values. The C-N distance used for the REDOR simulation is 1.51 Å, the best fitted value of Fig. 2(b).

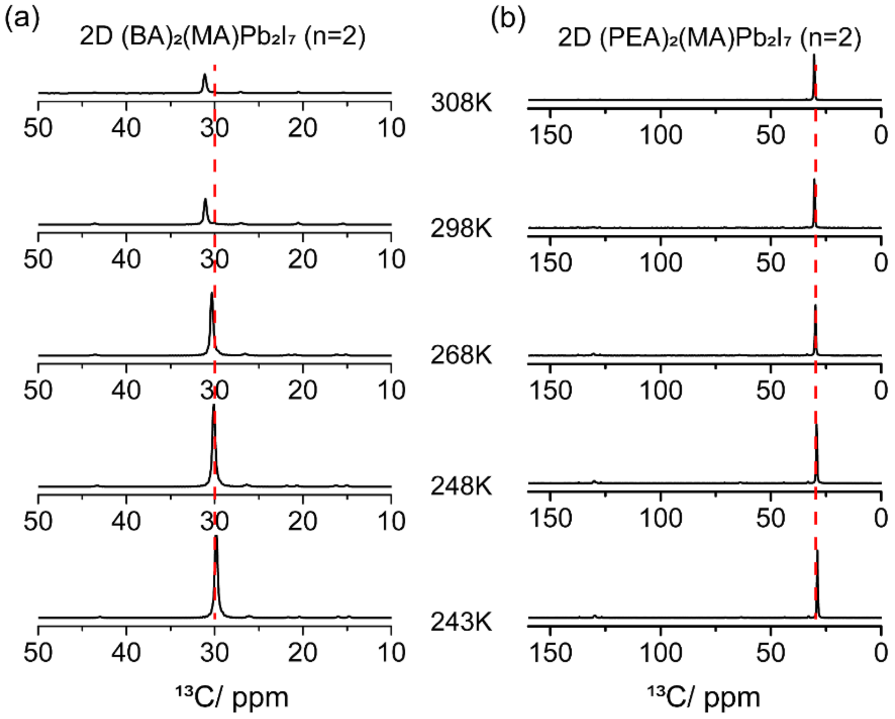

Supplementary Fig. 5 ^{13}C CPMAS NMR spectra of 2D OIHP crystals (n = 2). The full-scaled ^{13}C CPMAS spectra of (a) 2D $(BA)_2(^{13}C,^{15}N\text{-}MA)Pb_2I_7$ (n = 2) and (b) 2D $(PEA)_2(^{13}C,^{15}N\text{-}MA)Pb_2I_7$ (n = 2) recorded at various temperatures, ranging from 308 to 243K. The dashed lines are guides to the eye. The resonance peak of MA was found to have an upfield shift from 31.2 to 29.8 ppm with the temperature decreased from 308 to 243K.

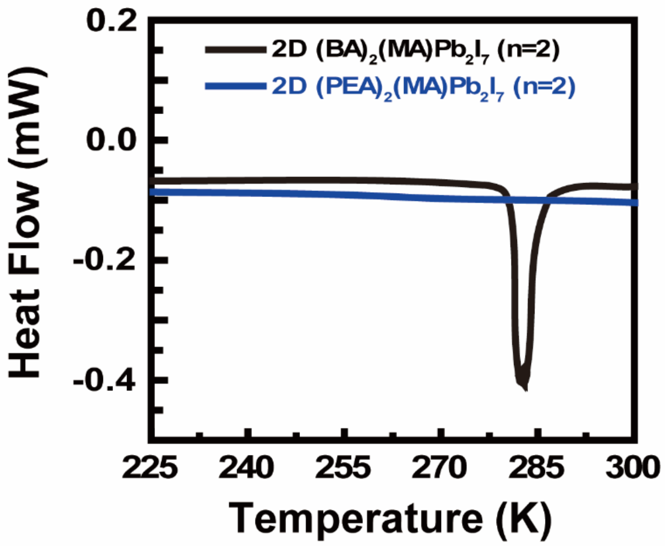

Supplementary Fig. 6 The differential scanning calorimetry (DSC) measurement of 2D $(BA)_2(MA)Pb_2I_7$ (n = 2, black) and 2D $(PEA)_2(MA)Pb_2I_7$ (n = 2, blue) crystals.

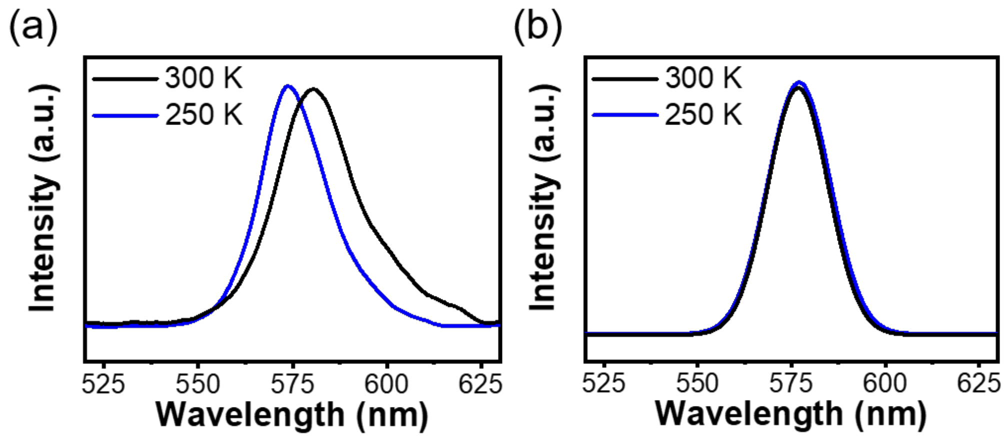

Supplementary Fig. 7 The PL spectra of (a) 2D $(BA)_2MAPb_2I_7$ (n = 2) and (b) 2D $(PEA)_2MAPb_2I_7$ (n = 2) recorded at 300K and 250K.

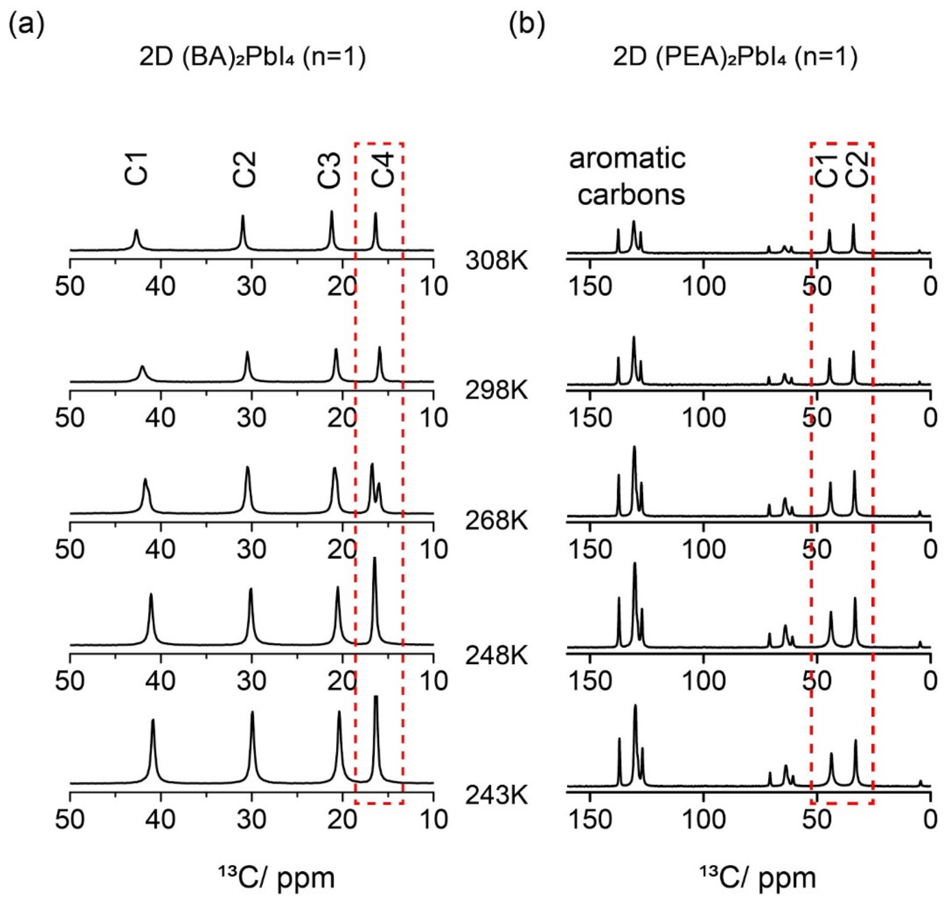

Supplementary Fig. 8 ^{13}C CPMAS NMR spectra of 2D OIHP crystals (n = 1). The temperature dependent ^{13}C spectra of (a) 2D $(BA)_2PbI_4$ (n = 1) and (b) 2D $(PEA)_2PbI_4$ (n = 1) recorded from 308K to 243K. The resonance peak of the C4 of BA in 2D $(BA)_2PbI_4$ (n = 1) was observed to split into two peaks as the temperature decreased from RT to 268 K, indicating a potential phase transition was occurred as reported previously[3]. As the temperature further decreased to 248K and 243K, only one peak of C4 of BA was observed. By contrast, there is no change in the chemical environment either at peak splitting or at peak position shift during the cooling process for 2D $(PEA)_2PbI_4$ (n = 1), consistent with a previous study where no phase change of 2D $(PEA)_2PbI_4$ (n = 1) was observed at this temperature range[4]. The dashed boxes are guides to the eye.

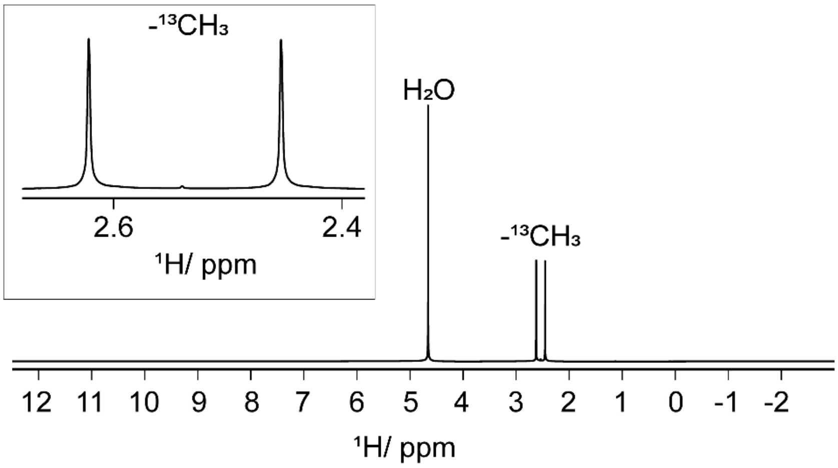

Supplementary Fig. 9 ^{1}H NMR spectrum of ^{13}C-methyl-^{15}N-amine iodide in D_2O (850.3 MHz). The methyl group ^{1}H-^{13}C *J*-coupling constant is 143 Hz.

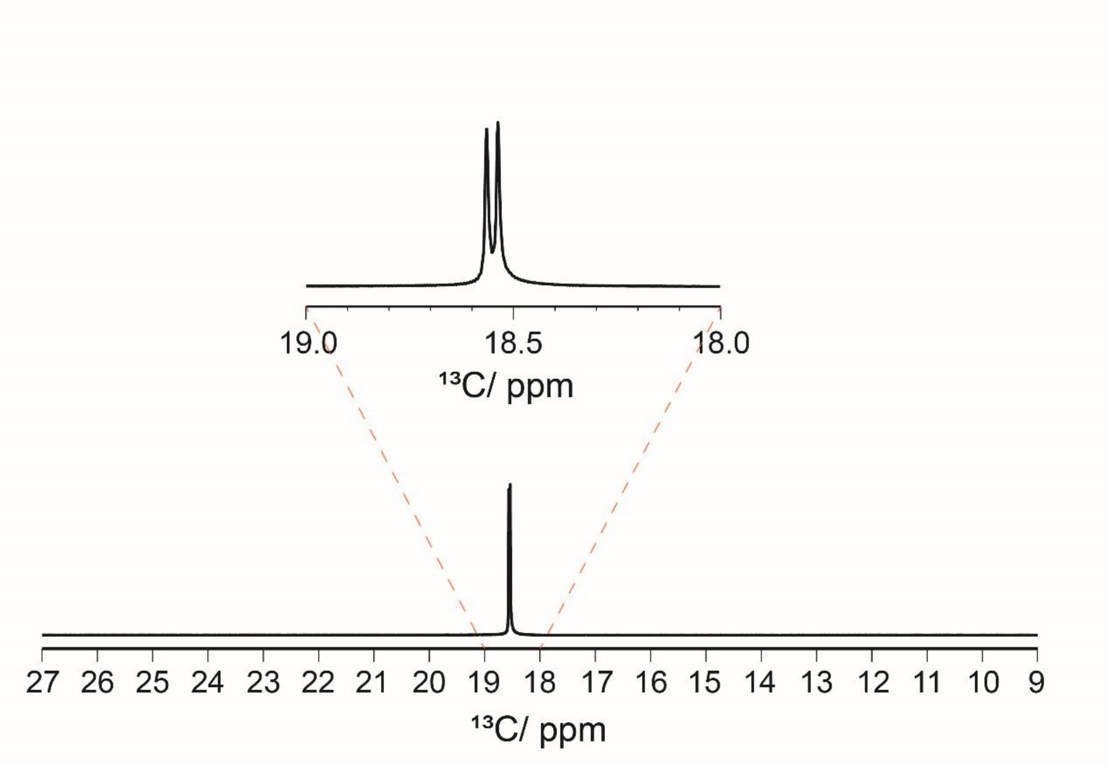

Supplementary Fig. 10 Proton decoupled ^{13}C-NMR spectrum of ^{13}C-methyl-^{15}N-ammonium iodide in D_2O (213.84 MHz). A ^{13}C-^{15}N J-coupling of 6 Hz is observed.

2. Supplementary References

1 Lipari, G. & Szabo, A. Model-free approach to the interpretation of nuclear magnetic resonance relaxation in macromolecules. 1. theory and range of validity. *J. Am. Chem. Soc* **104**, 4546-4559 (1982).

2 Haller, J. D. & Schanda, P. Amplitudes and time scales of picosecond-to-microsecond motion in proteins studied by solid-state NMR: a critical evaluation of experimental approaches and application to crystalline ubiquitin. *J. Biomol. NMR* **57**, 263-280 (2013).

3 Lyu, F. *et al.* Spatiodynamics, photodynamics, and their correlation in hybrid perovskites. *Chem. Mater* **33**, 3524-3533 (2021).

4 Chakraborty, R. & Nag, A. Correlation of dielectric confinement and excitonic binding energy in 2D layered hybrid perovskites using temperature dependent photoluminescence. *J. Phys. Chem. C* **124**, 16177-16185 (2020).
